# Nitrate Metabolism Modulates Biosynthesis of Biofilm Components in Uropathogenic *Escherichia coli* and Acts as a Fitness Factor During Experimental Urinary Tract Infection

**DOI:** 10.3389/fmicb.2020.00026

**Published:** 2020-01-31

**Authors:** Alberto J. Martín-Rodríguez, Mikael Rhen, Keira Melican, Agneta Richter-Dahlfors

**Affiliations:** ^1^Department of Neuroscience, Swedish Medical Nanoscience Center, Karolinska Institutet, Solna, Sweden; ^2^Department of Microbiology, Tumor and Cell Biology, Karolinska Institutet, Solna, Sweden; ^3^Laboratory for Molecular Infection Medicine Sweden (MIMS), Department of Molecular Biology, Umeå Centre for Microbial Research (UCMR), Umeå University, Umeå, Sweden

**Keywords:** nitrate respiration, biofilm, curli, tissue microbiology, uropathogenic *Escherichia coli*, urinary tract infection, *in vivo*

## Abstract

To successfully colonize a variety of environments, bacteria can coordinate complex collective behaviors such as biofilm formation. To thrive in oxygen limited niches, bacteria’s versatile physiology enables the utilization of alternative electron acceptors. Nitrate, the second most favorable electron acceptor after oxygen, plays a prominent role in the physiology of uropathogenic *Escherichia coli* (UPEC) and is abundantly found in urine. Here we analyzed the role of extracellular nitrate in the pathogenesis of the UPEC strain CFT073 with an initial focus on biofilm formation. Colony morphotyping in combination with extensive mutational, transcriptional, and protein expression analyses of CFT073 wild-type and mutants deficient in one or several nitrate reductases revealed an association between nitrate reduction and the biosynthesis of biofilm extracellular matrix components. We identified a role for the nitrate response regulator NarL in modulating expression of the biofilm master regulator CsgD. To analyze the role of nitrate reduction during infection *in vivo*, we tested wild-type CFT073 and a nitrate reductase null mutant in an ascending urinary tract infection (UTI) model. Individually, each strain colonized extensively, suggesting that nitrate reduction is expendable during UTI. However, during competitive co-infection, the strain incapable of nitrate reduction was strongly outcompeted. This suggests that nitrate reduction can be considered a non-essential but advantageous fitness factor for UPEC pathogenesis. This implies that UPEC rapidly adapts their metabolic needs to the microenvironment of infected tissue. Collectively, this work demonstrates a unique association between nitrate respiration, biofilm formation, and UPEC pathogenicity, highlighting how the use of alternative electron acceptors enables bacterial pathogens to adapt to challenging infectious microenvironments.

## Introduction

Bacteria are physiologically adaptable organisms that can establish stable communities in a multitude of environments (van Elsas et al., 2011; Retchless and Lawrence, 2012). Complex multicellular behaviors, such as biofilm formation, enable bacterial adaptation to environmental challenges (Kostakioti et al., 2013; Flemming et al., 2016). Bacterial biofilms form when adherent bacteria embed themselves in a self-produced extracellular matrix (ECM; Anderson et al., 2003). Biofilm formation on urinary catheters is one of the leading causes of nosocomial infections worldwide and has been linked to increased morbidity and mortality of urinary tract infections (UTIs; Flores-Mireles et al., 2015). Many *E. coli* strains can readily form biofilm on both abiotic and biotic surfaces, and they have been used as an archetypal model for biofilm research. Two major constituents of the *E. coli* biofilm ECM are curli fimbriae and cellulose (Bokranz et al., 2005). The biosynthesis of these components is regulated by the transcriptional regulator CsgD, which is central for environmental signal integration and a “master switch” for biofilm formation (Mika and Hengge, 2014).

A common environmental signal that bacteria encounter is oxygen limitation. Anaerobic physiology, including the use of alternative electron acceptors for respiration, is another important adaptive mechanism. Reduction of alternative electron acceptors has been shown to promote biofilm formation in clinical and environmental isolates of *Shewanella algae* (Martín Rodríguez, 2016), while oxygen has been found to foster biofilm lifestyles in *E. coli* (Bjergbaek et al., 2006; Eberly et al., 2017). With a versatile respiratory system, *E. coli* can use diverse electron acceptors for respiration including oxygen, dimethylsulfoxide (DMSO), *N*-trimethylamine oxide (TMAO), fumarate, nitrite, and nitrate. From an energetic standpoint, nitrate is second to oxygen (Cole, 1996). Recent studies have suggested that in *E. coli*, there is a strain-dependent association between terminal electron acceptor availability and biofilm formation (Bjergbaek et al., 2006; Eberly et al., 2017). *E. coli* can reduce nitrate for different purposes, such as a terminal electron acceptor (respiration), redox balancing (dissimilation), and growth (assimilation; Moreno-Vivian et al., 1999). Nitrate reduction in *E. coli* is performed by the terminal reductases NarGHJI, NarZYWV, and NapFDAGHBC, the first two being membrane-bound (respiratory reductases) and the latter, periplasmic (dissimilatory reductase). While the *narZYWV* operon is constitutively expressed, transcription of *narGHJI* and *napFDAGHBC* is tightly regulated by fumarate and nitrate regulator Fnr and the nitrate response regulators NarL and NarP depending on oxygen and nitrate availability (Schroder et al., 1993; Darwin et al., 1998; McNicholas and Gunsalus, 2002).

The mammalian urinary tract has inherent oxygen gradients, with the bladder considered to be a moderately oxygenated environment (Wang et al., 2008) and the kidney becoming increasingly hypoxic toward the medulla (Welch, 2006). This variation in oxygenation indicates that bacterial colonizers must be genetically equipped to take advantage of alternative electron acceptors in hypoxic microenvironments. Understanding how bacteria adapt to the rapidly changing infectious microenvironment is central to the concept of tissue microbiology, the study of infection in living tissue (Mansson et al., 2007; Melican et al., 2008, 2011). Using this approach, we previously described how uropathogenic *E. coli* (UPEC) virulence factors modulated bacterial fitness during infection in living kidneys (Melican et al., 2011). This work highlighted that while not strictly necessary for infection, fitness factors enhance the efficiency of tissue colonization *in vivo*. During kidney infection, we also observed the onset of vascular dysfunction within hours of infection, resulting in a rapid and local drop in tissue oxygen tension (Melican et al., 2008). This lack of oxygen at the site of infection suggests that the infecting bacteria must find alternative electron acceptors to adapt to this microenvironment. Nitrate is present in human urine at concentrations ranging from 0.25 to 2 mM depending on dietary uptake (Green et al., 1982; Tsikas et al., 1994). UPEC and other uropathogens can reduce urinary nitrate to nitrite, leading to high nitrite concentration in urine (nitrituria), a common marker for UTI. In this work, we focus on the physiology of nitrate reduction in the UPEC strain CFT073 outside and inside a living host. Through extensive genetics, we describe a unique association between nitrate reduction and biofilm ECM biosynthesis *in vitro*. We also identify nitrate reduction as a fitness factor *in vivo* that contributes to the effective colonization of the urinary tract during UTI.

## Methods

### Bacterial Strains and Growth Conditions

Bacterial strains used in this study are listed in Supplementary Table S1. Clinical UPEC strains used in biofilm tests have been published (Veses-Garcia et al., 2018). Mutants were prepared in *E. coli* CFT073 using the λ-red recombination system (Datsenko and Wanner, 2000; Yu et al., 2000). Gene disruptions were inspected by PCR using the “check” primer pairs (Supplementary Table S2). For genetic complementation, the genes and operons of interest were cloned with their native promoter regions in plasmid pGEN-MCS (Lane et al., 2007) using the primers listed in Supplementary Table S2. Epitope tagging of CsgD with a C-terminal 3XFLAG sequence (Uzzau et al., 2001) was performed using primers *csgD*-FLAG-F and *csgD*-FLAG-R (Supplementary Table S2). All constructs were confirmed by Sanger sequencing (Eurofins Genomics, Germany). Bacteria were routinely cultured at 37°C in LB broth (4 ml, 150 rpm) or LB agar supplemented with ampicillin (100 μg ml^−1^) or kanamycin (50 μg ml^−1^) when necessary. Unless otherwise stated, all chemicals were purchased from Sigma-Aldrich.

To analyze the growth characteristics of *E. coli* CFT073 wild-type (WT) and nitrate reduction null mutant (Supplementary Table S1), the OD_600_ of overnight cultures (LB, 37°C, 150 rpm) was first normalized to 1.0 and then diluted 1:100 in 20 ml LB. Growth curves (37°C, 150 rpm) of each strain separately or of a 1:1 co-culture of both strains were monitored by OD_600_ every 30 min. In parallel, CFU counts were determined on LB agar plates supplemented with kanamycin (50 μg ml^−1^) when appropriate to select for mutant CFUs.

### Nitrate Reductase Activity

Nitrate reductase activity of bacterial strains was determined by measuring nitrite accumulation in the supernatants of static cultures in LB medium (5 ml) supplemented with 20 mM sodium nitrate upon reaction with sulfanilamide and *N*-(1-naphthyl)ethylenediamine as previously described (Streeter and Devine, 1983). Optical readings were taken at 550 nm in 96-well plates (Tecan Infinite M1000 Pro). Experiments involved a minimum of three replicates.

### Congo Red Binding Assays

LB no salt (LBNS) agar plates were supplemented with Congo Red (CR, 40 μg ml^−1^) and Coomassie brilliant blue (20 μg ml^−1^). When indicated, sodium nitrate (0.2, 2 or 20 mM), ampicillin (100 μg ml^−1^), and/or arabinose (0.1%) were supplemented to the agar. A 10 μl cell suspensions (OD_600_ = 1.0) of the relevant strains were spotted on the agar and allowed to dry, and plates were incubated at 28°C for 48 h unless otherwise indicated before colony morphologies were monitored.

### Calcofluor Binding Assays

LBNS agar plates were supplemented with calcofluor white (50 μg ml^−1^) and, when indicated, sodium nitrate (20 mM). A 10 μl cell suspensions (OD_600_ = 1.0) of the relevant strains were spotted on the agar and allowed to dry, and plates were incubated at 28°C for 48 h. Calcofluor binding on colonies was revealed under UV light.

### Motility Tests

Swimming motility was assessed in soft agar (1% tryptone, 0.5% NaCl, and 0.25% agar) supplemented with sodium nitrate (20 mM) when indicated. An inoculation needle was dipped into overnight cultures normalized to an OD_600_ of 1.0 and used to stab the center of the plates, which were incubated at 30°C for 16 h before reading.

### Cyclic Diguanosine Monophosphate Analysis

For cyclic diguanosine monophosphate (c-di-GMP) turnover protein overexpression, the genes encoding the diguanylate cyclase Slr1143 from *Synechocystis* sp. PCC6803 and the c-di-GMP-specific phosphodiesterase YhjH from *E. coli* CFT073 were cloned in plasmid pBAD/Myc-His using the primers listed in Supplementary Table S2. Plasmid pBAD-*slr1143* (Supplementary Table S2) and genomic DNA (gDNA) from *E. coli* CFT073 were used as DNA templates. Gene expression was induced by the addition of 0.1% arabinose. To obtain an indirect estimation of the total intracellular [c-di-GMP], the riboswitch biosensor pRP0122-P*be-amcyan_Bc3-5_turborfp* (Zhou et al., 2016) was employed (Supplementary Table S2). Relative Amcyan/TurboRFP fluorescence ratios were determined spectrophotometrically at 489 nm (Amcyan) and 574nm (TurboRFP) as described (Zhou et al., 2016).

### RNA Isolation and Quantitative Real-Time Polymerase Chain Reaction

Bacterial colonies were grown from 10 μl spots as described above on LBNS plates with 0 or 20 mM nitrate at 28°C for 15 h. Each colony was developed from an independent culture. For RNA isolation, colonies were individually scraped from the plates, re-suspended in 600 μl of TRI-Reagent (Zymo Research), and mechanically disrupted in Lysing Matrix B tubes containing 0.1 mm silica beads (MP Biomedicals). RNA extraction was performed using the Direct-Zol RNA Miniprep Plus (Zymo Research), and residual gDNA was removed by DNase treatment using the Turbo DNA-free kit (Ambion). Complementary DNA was synthesized with the Superscript III First-Strand Synthesis Supermix for qRT-PCR (Invitrogen) using 1 μg of total RNA. Quantitative PCR reactions using primers listed in Supplementary Table S2 were conducted in the LightCycler 480 System (Roche) using the pre-defined SYBR Green reaction protocol. Relative gene expression was calculated by the ΔΔCt method using *rpoD* expression for normalization.

### Western Blots

Single colonies were lysed in SDS sample buffer. Proteins were separated on pre-cast 4–12% Bis-Tris SDS-PAGE gels (NuPAGE) and transferred to 0.2 μm PVDF membranes. Blots were probed with anti-FLAG antibody (1:1000, Sigma-Aldrich), anti-RecA antibody (1:6000, Amersham), or anti-RpoS antibody (1:750, Biolegend) and HRP-conjugated secondary antibodies (1:10000, goat anti-rabbit, Invitrogen or sheep anti-mouse, GE Healthcare). Blots were developed with the ECL Prime Western Blotting Detection Reagent (Amersham) and imaged (GelDoc XRS^+^). Relative protein quantities were determined with Image Studio Lite (LIC-OR Biosciences) using RecA levels for normalization. Three replicates were performed.

### *In vivo* Infection Experiments

Animal experimental protocols were performed in accordance with the Swedish Board of Agriculture’s Regulations and General Advice on Laboratory Animals (L150) and were approved by the Stockholms Norra Djurförsöksetiska Nämnd (permit number N34/14).

Ascending UTI was performed as previously described (Melican et al., 2011). Briefly, anesthetized (isoflurane) female Sprague-Dawley rats (231 ± 13 g) were inoculated intra-urethrally with 100 μl bacterial suspensions in PBS, resulting in a total infectious dose of 10^8^ CFU of *E. coli* strains CFT073 WT, a nitrate reduction null mutant or a 1:1 mix of these two strains. Sham-infected controls were intra-urethrally infused by 100 μl sterile PBS. Animals were housed with free access to water and chow. Animals were sacrificed after 4 days by exsanguination under anesthesia, urinary bladder and kidneys were dissected, and urine and blood samples were taken. The organs were placed in 0.5 ml PBS buffer and homogenized (FastPrep-24, MP Biomedicals). CFU counts from the homogenates as well as from the urine and blood were determined using LB agar supplemented with Km (50 μg ml^−1^) when necessary.

## Results

### *E. coli* CFT073 Biofilm Morphotypes Are Modulated by Membrane-Bound Nitrate Reductases

**Figure 1:**
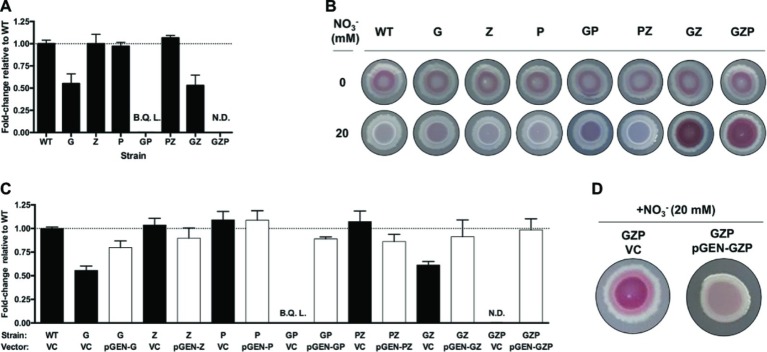
Nitrate respiration determines colony biofilm morphotypes in *E. coli* CFT073. **(A)** Nitrite production in nitrate-supplemented (20 mM) static cultures of *E. coli* CFT073 WT and nitrate reductase mutants G, Z, P, GP, PZ, GZ, and GZP, reported as fold-change relative to the WT normalized to 1 (dotted line). B.Q.L. = Below Quantification Limit, representing >1,000-fold lower than WT levels. N.D. = Not Detected. Data represent the mean and standard ldeviation of a minimum of three replicates. **(B)** Colony morphotypes of *E. coli* CFT073 WT and nitrate reductase mutants at 0 (upper row) and 20 mM (lower row) nitrate. The colonies were grown on LBNS agar plates at 28°C for 48 h. **(C)** Nitrite production in nitrate-supplemented (20 mM) static cultures of *E. coli* CFT073 WT and mutants complemented with the empty plasmid pGEN-MCS as vector control (VC, black bar) or vectors carrying the corresponding operons (pGEN-G, -Z, -P, -GP, -PZ, -GZ, -GZP, white bar). Results are reported as fold-change relative to WT VC normalized to 1 (dotted line). B.Q.L. = Below Quantification Limit, representing >1,000-fold lower than WT levels. N.D. = Not Detected. Data represent the mean and standard deviation of a minimum of three replicates. **(D)** Colony morphotypes showing phenotypic complementation of the GZP nitrate reduction null mutant harboring the vector control (VC) or the complementing plasmid pGEN-GZP, in which the three deleted operons are cloned in tandem under their respective native promoters.

To analyze the effect of nitrate reduction on biofilm formation in *E. coli* CFT073, we prepared isogenic mutants lacking the membrane-bound and periplasmic nitrate reductases. Deletion of the operons encoding the nitrate reductases NarGHJI, NarZYWV (both membrane-bound), and NapFDAGHBC (periplasmic) resulted in the three single mutants Δ*narGHJI*, Δ*narZYWV*, and Δ*napFDAGHBC*, which we hereafter refer to as G, Z, and P, respectively. We also prepared the corresponding GP, PZ, and GZ double mutants, as well as the triple mutant GZP (Supplementary Table S1). To determine nitrate reductase activity in WT and mutant strains, we measured nitrite accumulation in the supernatant of nitrate-supplemented static cultures (Figure 1A). Compared to the WT, the G mutant displayed a 50% reduction in nitrate reductase activity, while Z and P remained at WT levels. In the GP double mutant, nitrite production was three orders of magnitude lower than the WT, the GZ mutant showed 50% reduction, and the PZ mutant remained at WT levels. In the GZP triple mutant, no nitrite production was detected. Collectively, these results show that the NarGHJI reductase is most critical for nitrate reduction. Furthermore, the combination of NarGHJI and NapFDAGHBC is essential for proficient nitrate reduction to occur under our experimental conditions.

Next, we tested whether these mutations had any influence over biofilm formation. Using the colony biofilm model based on CR-containing agar plates, we analyzed the biofilm morphotype of WT and mutant strains growing in the absence and presence of nitrate (20 mM). Testing these strains under anaerobic conditions resulted in poor growth, which prevented further analysis in the absence of oxygen *in vitro* (data not shown). Under standard aerobic conditions without nitrate, all the strains exhibited the same **s**mooth **a**nd **r**ed (*sar*) morphotype (Figure 1B). Upon nitrate supplementation, the WT became **s**mooth **a**nd **w**hite (*saw*) suggesting decreased production of CR-binding compounds in the ECM. This phenotype remained similar in mutants G, Z, P, GP, and PZ, with minor changes in the colony center. By contrast, mutants GZ and GZP exhibited a more intense *sar* morphotype upon nitrate supplementation, which was more evident in the colony center. When comparing nitrate reductase activity (Figure 1A) and colony phenotypes (Figure 1B), we deduced that the activities of the membrane-bound reductases NarGHJI and NarZYWV determined the colony morphotype in the presence of nitrate under the tested conditions.

To verify that the observed *sar* and *saw* morphotypes indeed originated from mutations in the reductases rather than as a consequence of polar or secondary mutations, we performed genetic complementation. The *narGHJI*, *narZYWV*, and *napFDAGHBC* operons were cloned individually and in tandem with their native promoter regions into the low-copy number plasmid pGEN-MCS. This resulted in a set of complementation vectors defined as pGEN-G, pGEN-Z, pGEN-P, pGEN-GP, pGEN-PZ, pGEN-GZ, and pGEN-GZP, with the empty plasmid serving as vector control. Contrary to the respective strain transformed with the vector control, ectopic expression of the deleted operons in all mutant strains restored nitrate reductase activity to levels exceeding 75% that of the WT strain (Figure 1C). Restoration of the nitrate reductase activity correlated also to a phenotypic complementation, here shown for the triple mutant GZP, which upon complementation reverted from the *sar* to the *saw* morphotype on nitrate supplemented CR agar (Figure 1D).

To study whether the observed changes in colony biofilm in response to nitrate was common among UPEC strains, we evaluated the colony biofilm morphotypes in a collection of 40 clinical UPEC isolates (Veses-Garcia et al., 2018). A palette of strain dependent phenotypes was seen when grown on CR LBNS agar in the absence or presence of 20 mM nitrate, with 22 of 40 demonstrating a decrease in CR binding and/or colony roughness (Supplementary Figure S1). Taken together, this reveals that the association between nitrate reduction and biofilm formation in *E. coli* CFT073 is not exclusive to this strain but shared by more than half of the clinical UPEC strains tested. Moreover, our experiments with *E. coli* CFT073 suggest that it is respiratory nitrate reduction, not merely the ability to reduce nitrate *per se*, that triggers a switch in colony biofilm structure upon nitrate supplementation.

### Bacterial Motility Is Not Influenced by Exogenous Nitrate

**Figure 2:**
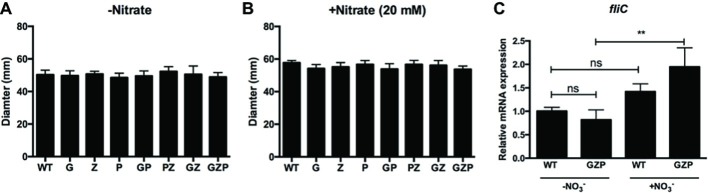
Swimming motility is independent of nitrate availability. **(A,B)** Diameter of swimming halos of *E. coli* CFT073 WT and nitrate reductase mutants G, Z, P, GP, PZ, GZ, and GZP in the absence **(A)** and presence **(B)** of 20 mM nitrate. Data show the mean and standard deviation of ≥6 independent experiments. **(C)** Relative mRNA expression of *fliC* in WT and GZP colony biofilms grown in the absence and presence of 20 mM nitrate for 15 h at 28°C. Data show the mean and standard deviation of three replicates. Statistical significance is determined by one-way ANOVA followed by Dunnett’s *post hoc* test (**p ≤ 0.01; ns = *p* > 0.05).

In *E. coli,* biofilm formation and motility are generally inversely expressed (Pesavento et al., 2008). We therefore investigated whether the changes in the observed colony morphotypes correlated to any changes in the swimming ability. Motility assays of the single, double, and triple nitrate reductase mutants displayed no alteration of the swimming halos compared to the WT, irrespective of nitrate addition to the soft agar (Figures 2A,B). Since swimming motility involves the expression of flagellar genes, we quantified the transcript levels of *fliC* that encodes the major structural protein flagellin. In colony biofilms of the WT that express full nitrate reduction capacity, nitrate supplementation did not significantly increase *fliC* transcript levels (Figure 2C). In the GZP triple mutant that represents a nitrate reduction null strain, a statistically significant increase in 1.9-fold was observed upon the addition of nitrate. As no biological effect was observed in *fliC* expression and swimming motility, we concluded that flagellar-based motility is independent of the inverse phenotypic switch observed in colony biofilms of the WT and nitrate reductase mutants in response to exogenous nitrate.

### Diguanylate Cyclase Overexpression Reveals Inverse Regulation of Extracellular Matrix Components by Nitrate

**Figure 3:**
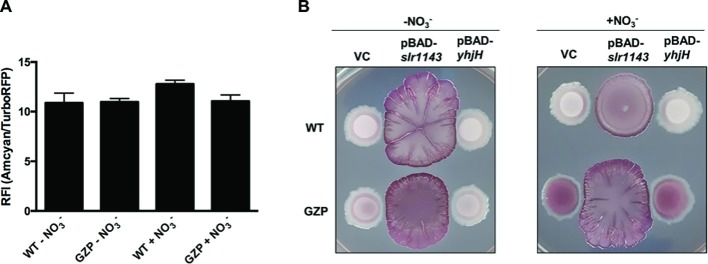
High [c-di-GMP] reveals nitrate respiration-mediated inverse regulation of biofilm ECM components. **(A)** Relative fluorescence intensity (RFI) showing the ratio between green and red fluorescence in colony biofilms formed by WT and GZP harboring the c-di-GMP riboswitch reporter plasmid pRP0122-P*be*-*amcyan*_*Bc3*-*5*_*turborfp*. Data from colony biofilms grown for 48 h at 28°C on LBNS in the absence or presence of 20 mM nitrate are shown as indicated. Data show the mean and standard deviation of three replicates. **(B)** Colony morphotypes of WT (upper row) and the GZP mutant (lower row) overexpressing the diguanylate cyclase Slr1143 (pBAD-*slr114*3) and the c-di-GMP phosphodiesterase YhjH (pBAD-*yhjH*) when grown in the absence and presence of 20 mM nitrate. Strains harboring the empty plasmid pBAD-Myc/His, used for cloning, serve as vector control (VC).

Biofilm formation in *E. coli* is mediated by the second messenger c-di-GMP. High [c-di-GMP] promotes biofilm formation, whereas low [c-di-GMP] fosters motile behaviors (Romling et al., 2013). To estimate the total [c-di-GMP] in colony biofilm cells of the WT and the nitrate reduction null mutant strain GZP, we used a c-di-GMP riboswitch reporter plasmid. This reporter generates red fluorescence from cells with high intracellular [c-di-GMP], while cells with low concentration fluoresce in green (Zhou et al., 2016). When the green-to-red fluorescence ratios were analyzed in WT and GZP colony biofilm cells spectrophotometrically, no significant difference was observed between strains, nor between cells growing in the absence or presence of nitrate (Figure 3A). Thus, as measured by the reporter construct, we could not demonstrate any major alterations in the global pool of c-di-GMP.

To study how exogenous nitrate may affect cells with high and low [c-di-GMP], we artificially altered the c-di-GMP pools in WT and GZP cells. Overexpression of the diguanylate cyclase (DGC) Slr1143 leads to increased [c-di-GMP], while overexpression of the c-di-GMP-specific phosphodiesterase (PDE) YhjH is expected to lead to low c-di-GMP pools. Analysis of the biofilm morphotype of the WT overexpressing YhjH showed that low [c-di-GMP] did not influence the colony phenotypes, irrespective of nitrate presence (Figure 3B). Similarly, low [c-di-GMP] did not influence the phenotypic switch of GZP upon nitrate supplementation. Overexpression of Slr1143 induced an exacerbated **r**ed, **d**ark, **a**nd **r**ough (*rdar*) morphotype in both genetic backgrounds in the absence of nitrate. Nitrate addition attenuated this morphotype in the WT colony biofilm, which exhibited reduced size and round shape with defined margins. The GZP *slr1143*^+^ colony displayed even higher roughness and irregular margins in the presence of nitrate than in the absence of it (Figure 3B). As these phenotypes are typically attributed to overproduction of curli and cellulose, our data suggest a correlation between environmental nitrate, nitrate respiration, and the production of biofilm ECM components under the tested conditions.

### Nitrate Sensors and Regulators Modulate CsgD-Dependent Extracellular Matrix Biosynthesis in *E. coli* Colony Biofilms

**Figure 4:**
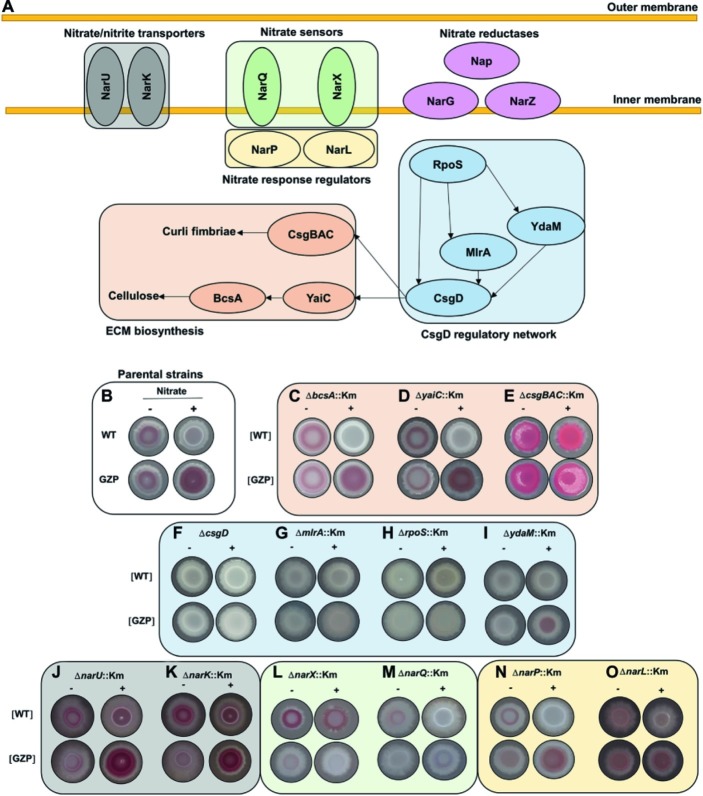
Mutational analysis to probe for a regulatory role of environmental nitrate in biofilm formation. **(A)** Schematic representation of key proteins involved in nitrate transport (NarU, NarK), nitrate sensing (NarQ, NarX), cognate response regulators (NarP, NarL), and nitrate reduction. The CsgD regulatory network includes RpoS, YdaM, MlrA, and CsgD, with arrows indicating known network connections. This network controls biofilm ECM biosynthesis through the expression of curli fimbriae (CsgBAC) and cellulose (YaiC, BcsA). **(B)** Phenotypes of *E. coli* CFT073 WT (upper row) and GZP mutant (lower row), referred to as parental strains, in the absence (−) and presence (+) of 20 mM nitrate, reproduced from Figure 1. **(C–O)** Colony morphotypes of indicated isogenic mutants, prepared in the WT and GZP genetic backgrounds ([WT] and [GZP]), grown in the absence (−) and presence (+) of 20 mM nitrate. The colonies were grown for 48 h at 28°C on LBNS medium. Images are arranged and color coded according to the functional groups shown in **(A)**.

In most *E. coli* strains, the biosynthesis of curli and cellulose is controlled by CsgD, whose expression is integrated in a highly complex and well-characterized regulatory network (Serra and Hengge, 2014). Likewise, nitrate respiration is controlled by a finely tuned network of transporter proteins, sensors, and cognate response regulators (Rabin and Stewart, 1993; Jia and Cole, 2005). To identify a possible genetic association between nitrate transport, sensing, metabolism, respiration, and ECM biosynthesis, we performed a systematic mutational analysis based on the WT and GZP genetic backgrounds, hereafter referred to as [WT] and [GZP], in which gene knockouts were prepared for all of the genes indicated in Figure 4A.

The colony phenotypes of the parental strains WT and GZP (Figure 4B) were used as references when examining the morphotypes of the mutant strains on CR-containing agar plates in the presence or absence of nitrate. To analyze the role of the ECM biosynthesis genes, we knocked out the genes encoding curli fimbriae (*csgBAC*), the cellulose synthase (*bcsA*), and the diguanylate cyclase *yaiC,* which regulate BcsA activity *via* local production of c-di-GMP. The Δ*bcsA*::Km and Δ*yaiC*::Km strains showed no difference compared to the WT and GZP parental strains, respectively (Figures 4C,D). By contrast, deletion of *csgBAC* resulted in the development of a pink and rough morphotype in both genetic backgrounds in the absence of nitrate (Figure 4E). Nitrate supplementation promoted a morphotypic switch of the [WT] Δ*csgBAC*::Km strain to smooth and pink, whereas the [GZP] Δ*csgBAC*::Km mutant strain showed increased roughness (Figure 4E). Collectively, this suggests curli fimbriae as the major contributor to the observed phenotypes. To confirm this observation, we performed calcofluor binding assays, a dye known to bind cellulose, with the WT and GZP strains and their respective Δ*bcsA*::Km and Δ*csgBAC*::Km isogenic mutants in the absence or presence of nitrate (Supplementary Figure S2). In the absence of nitrate (Supplementary Figure S2A), WT and GZP colony biofilms showed negligible calcofluor binding, similar to both Δ*bcsA*::Km mutants. By contrast, Δ*csgBAC*::Km mutants in both genetic backgrounds exhibited exacerbated calcofluor binding, indicating that the pink morphotype on CR agar was due to cellulose overproduction. These patterns remained similar when the test was performed in nitrate-supplemented agar (Supplementary Figure S2B), with the only exception of the GZP strain, which showed an incipient accumulation of calcofluor around the colony center. Altogether, this demonstrates that the main contribution to the observed colony biofilm morphotypes in *E. coli* CFT073 comes from curli biogenesis and suggests that the biosynthesis of other biofilm ECM components such as cellulose may be also regulated by nitrate in strains presenting more proficient biosynthesis.

Expression of *yaiC* and *csgBAC* is controlled by the CsgD regulatory network, in which the DNA-binding regulator CsgD acts as a master switch (Ogasawara et al., 2011). Transcription of *csgD* is controlled by the joint action of the transcription factor MlrA and the DGC YdaM, and ultimately, by the global stress response regulator RpoS (Figure 4A; Mika and Hengge, 2014). Analysis of the colony morphotypes formed by the Δ*csgD*::Km, Δ*mlrA*::Km, and Δ*rpoS*::Km mutants resulted in identical *saw* morphotypes regardless of the WT or the GZP genetic background and nitrate availability (Figures 4F–H). The Δ*ydaM*::Km mutant showed a highly attenuated *sar* morphotype (Figure 4I). Collectively, our results indicate that the colony morphotypes in the parental strains are CsgD-dependent.

Bacterial uptake of nitrate and nitrite, as well as nitrite extrusion, is performed by the transmembrane proteins NarU and NarK (Figure 4A; Clegg et al., 2002). To analyze the influence of these nitrate/nitrite transporters, we examined colony morphology in mutant strains lacking either of the two genes. Both the Δ*narU*::Km and Δ*narK*::Km mutants attenuated the *sar* phenotype in the absence of nitrate in the WT and GZP genetic backgrounds, whereas colony morphotypes remained similar to those of the parental strains in the presence of nitrate, with slightly increased CR binding in the WT background (Figures 4J–K). This suggested that nitrate/nitrite transport influences ECM biosynthesis to some extent but is not determinant for the divergent phenotypic switch in the WT and GZP strains upon nitrate addition.

Nitrate sensing and response regulation is governed by the two-component systems NarXL and NarQP (Figure 4A). In the WT, deletion of *narX* did not influence the colony morphotype in the absence of nitrate, whereas a morphotypic change was observed in the presence of nitrate, demonstrating an increased biosynthesis of biofilm ECM components (Figure 4L). Deletion of *narQ* in the WT background attenuated ECM production in the absence of nitrate, while the *saw* morphotype remained unchanged in the presence of nitrate (Figure 4M). When these mutations were introduced in the GZP background, deletion of either of the sensors resulted in *saw* colony morphotypes regardless of nitrate supplementation (Figures 4L,M). Next, we analyzed the effect of the nitrate response regulators. Deletion of *narP* in the [WT] and the [GZP] genetic backgrounds caused minor changes in the colony morphotypes irrespective of the absence or presence of nitrate (Figure 4N). Deletion of *narL*, however, greatly attenuated the *sar* phenotype with and without nitrate (Figure 4O). The phenotype of the parental WT and GZP strains could be restored by ectopic expression of NarL (Supplementary Figure S3). In summary, this mutational analysis suggests an interplay between nitrate sensing and respiration, resulting in a CsgD-dependent regulation of biofilm ECM biosynthesis in response to environmental nitrate. In this complex network, NarL-mediated gene expression appears to attenuate CsgD-driven ECM biosynthesis.

### NarL Promotes *csgD* and *csgA* Gene Expression in a Nitrate Reduction Null Mutant

**Figure 5:**
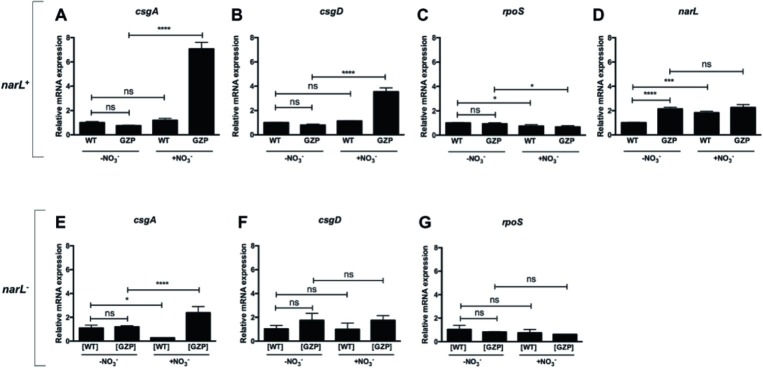
Transcriptional profiles of key genes relating nitrate respiration with biosynthesis of biofilm ECM components in *narL*^+^ and *narL*^−^ genetic backgrounds. **(A–D)** Upper panel shows relative mRNA expression of *csgA* **(A)**, *csgD* **(B)**, *rpoS* **(C)**, and *narL* **(D)** in colony biofilms of WT and nitrate reduction null mutant GZP in the absence or presence of 20 mM nitrate. **(E–G)** Lower panel shows relative mRNA expression of *csgA* **(E)**, *csgD* **(F)**, and *rpoS* **(G)** in [WT] Δ*narL*::Km and [GZP] Δ*narL*::Km. Data represent mean and standard deviation of three replicates. Statistical significance was determined by one-way ANOVA followed by Dunnett’s *post hoc* test (****p ≤ 0.0001; *p ≤ 0.05; ns = *p* > 0.05).

To dissect the transcriptional events linking nitrate sensing and reduction and the biosynthesis of ECM components, we analyzed the expression levels of the key genes *csgA*, *csgD*, and *rpoS* in WT and the GZP nitrate reduction null mutant colony biofilms. Transcript levels of *csgA*, encoding the major curlin subunit, remained constant in the WT irrespective of nitrate, while a significant increase was observed in the GZP mutant colony biofilm upon nitrate supplementation (Figure 5A). The same pattern was observed for the c*sgD* transcriptional profile (Figure 5B). Transcript levels of *bcsA*, which encodes the cellulose synthase, remained constant irrespective of the strain background or nitrate supplementation (Supplementary Figure S4), supporting a higher input from curli fimbriae with respect to cellulose on the observed phenotypes on CR. Transcript levels of *rpoS* were 0.3-fold lower in both WT and GZP colony biofilms in the presence of nitrate compared to the parental strains without nitrate (Figure 5C).

Based on the observed attenuated effect of *narL*^−^ mutants on ECM biosynthesis, we analyzed transcript levels of this response regulator. Expression of *narL* was significantly upregulated by environmental nitrate in WT colony biofilms (Figure 5D). Similarly, a significant increase in transcript levels was observed in the GZP mutant compared to the WT in the absence of nitrate. This elevated expression in the GZP mutant remained after the addition of nitrate (Figure 5D). This indicates that exogenous nitrate cannot modulate *narL* levels in the GZP mutant.

To further analyze the role played by NarL, we measured the expression of *csgA, csgD,* and *rpoS* in colony biofilms of the *narL*^−^ mutant strains [WT] Δ*narL*::Km and [GZP] Δ*narL*::Km. Exogenous nitrate altered the expression of *csgA* in both the [WT] Δ*narL*::Km and [GZP] Δ*narL*::Km (Figure 5E). The expression levels were, however, substantially lower than the equivalent experiments in *narL*^+^ background. Conversely, mRNA levels of *csgD* (Figure 5F) and *rpoS* (Figure 5G) remained approximately the same in all strain backgrounds, regardless of nitrate supplementation. For *csgD*, this result contrasts with the equivalent experiments in *narL*^+^ backgrounds, where nitrate supplementation induced significant changes in expression levels. Collectively, our results indicate that NarL contributes, directly or indirectly, to the transcription of *csgD* and hierarchically downstream genes.

### NarL Modulates CsgD Protein Expression in Response to Nitrate Reduction and Environmental Nitrate Levels

**Figure 6:**
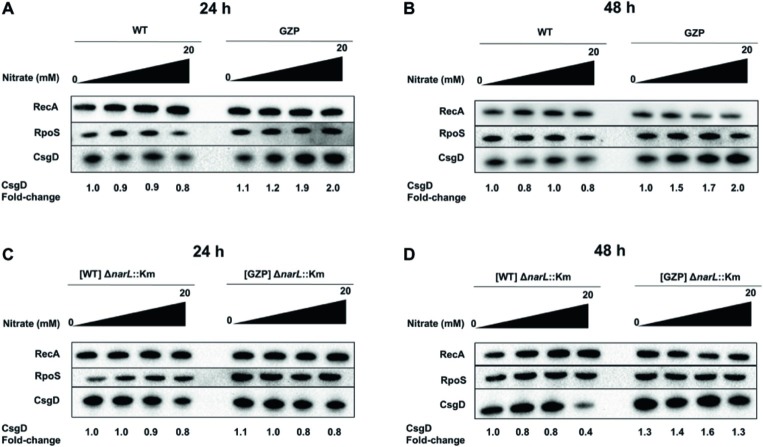
Effect of extracellular nitrate on protein expression of RpoS and CsgD in *narL*^+^ and *narL*^−^ strains. **(A,B)** Western blot showing RecA, RpoS, and CsgD in WT and GZP colony biofilms incubated for 24 h **(A)** and 48 h **(B)** at 28°C when exposed to 0, 0.2, 2, and 20 mM nitrate. **(C,D)** Western blots performed in [WT] Δ*narL*::Km and [GZP] Δ*narL*::Km after 24 h **(C)** and 48 h **(D)**. The housekeeping protein RecA is used as a loading control and for quantification of the relative amounts of CsgD, expressed as the average fold-change relative to the WT and [WT] Δ*narL*::Km colony biofilm with no nitrate supplementation (*N* = 3).

To investigate whether CsgD protein expression is influenced by the *narL* genotype, we prepared strains for Western blot analysis by introducing a 3XFLAG tag at the C-terminus of *csgD* in the WT and the GZP nitrate reduction null mutant, as well as in the corresponding [WT] Δ*narL*::Km and [GZP] Δ*narL*::Km strains. The 3XFLAG tag did not affect CsgD activity as determined by colony biofilm morphotyping (Supplementary Figure S5). Western blots showed that protein levels of CsgD in the WT strain remained constant in 24 h colony biofilms grown in the absence or presence of 0.2, 2, and 20 mM nitrate (Figure 6A). By contrast, elevated expression levels were observed in the GZP mutant exposed to increasing extracellular nitrate concentrations, reaching two-fold compared to the WT. The same patterns were maintained in 48 h colony biofilms (Figure 6B). When analyzing the CsgD expression level in the *narL*^−^ mutant backgrounds, at 24 h the level remained relatively stable with increasing nitrate concentration in the [WT] Δ*narL*::Km strain as well as the [GZP] Δ*narL*::Km (Figure 6C). In 48 h old colony biofilms, the [WT] Δ*narL*::Km strain showed a decrease in CsgD expression with increasing nitrate, while expression in the [GZP] Δ*narL*::Km strain remained stable (Figure 6D). To analyze whether the *narL* genotype involved any differential RpoS expression, we used a commercial antibody to determine the expression levels of this protein in *narL*^+^ and *narL*^−^ strains. Western blots showed that irrespective of the *narL* genotype, RpoS was expressed at similar levels in 24 and 48 h colony biofilms grown in the absence or the presence of nitrate (Figures 6A–D). Altogether, these experiments show that NarL differentially modulates CsgD protein levels in WT and GZP strains dependent on extracellular nitrate concentration, thereby contributing to the regulation of biofilm ECM biogenesis.

### Nitrate Reduction Provides a Fitness Advantage During Urinary Tract Infection

**Figure 7:**
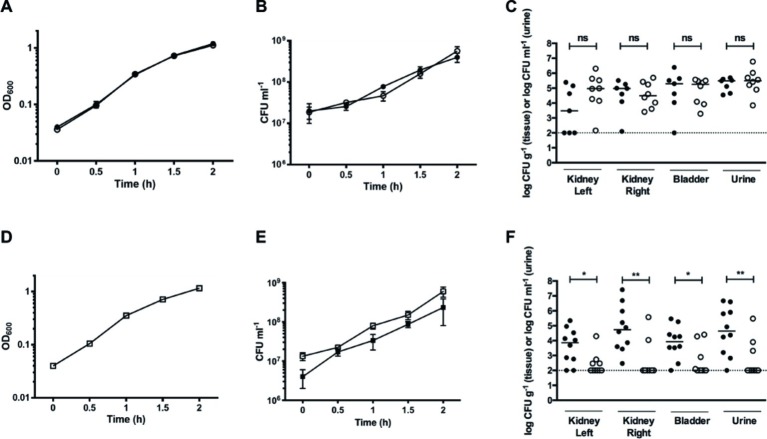
Nitrate reduction constitutes a fitness factor for UPEC *in vivo*. **(A)** Growth curves of individual cultures of WT (filled circles) and GZP harboring a Km cassette at the *narGHJI* locus (open circles). **(B)** CFU counts for individual cultures of WT (filled circles) and GZP-Km (open circles). **(C)** Bacterial load in kidneys, bladders, and urine of infected rats at 4 days post-infection following independent challenge with the WT (filled circles) and GZP-Km (open circles) strains. **(E)** Growth curve of WT and GZP-Km in mixed 1:1 co-culture. **(D)** CFU counts of the 1:1 mixed co-culture of WT:GZP-Km, showing total CFU counts (open squares) and GZP-Km (filled squares). **(F)** Bacterial load in kidneys, bladders, and urine of infected rats at 4 days post-infection after challenge with a mixed inoculum containing 1:1 WT (filled circles): GZP-Km (open circles). Bars in **(C)** and **(F)** indicate the median, and statistical significance is determined by the Mann-Whitney test (*p < 0.05; **p < 0.01; ns = *p* > 0.05). The dotted line indicates the CFU detection limit.

Nitrate is the second most energetically favorable electron acceptor, after oxygen. Nitrate metabolism is deeply integrated into the biology of UPEC, and the urinary tract contains niches abundant in nitrate (Green et al., 1982; Tsikas et al., 1994). Thus, we analyzed the role of nitrate reduction on UPEC pathogenesis *in vivo* during urinary tract infection. No growth rates differences were seen between WT and the GZP-Km nitrate reduction null mutant strains (Supplementary Table S1) in separate aerobic cultures *in vitro* (Figures 7A,B; Supplementary Figure S6). We therefore used these strains to investigate the impact of abrogated nitrate reductase activity on UPEC pathogenesis in the ascending UTI model. Female rats were trans-urethrally infected with either the WT or GZP-Km strains. Four days post-infection, we determined the CFU of urine, bladder, and each kidney. Equal numbers of both WT and the GZP-Km mutant were recovered from all sites (Figure 7C). This showed that the ability to reduce nitrate is not required for UPEC pathogenesis *in vivo*.

To study whether nitrate reduction may convey a fitness advantage during infection, we tested the GZP-Km mutant in direct competition with the WT strain. Initially, we analyzed growth curves of a 1:1 mixture of WT and GZP-Km in LB medium. Each strain was recovered in equal numbers throughout the growth experiment (Figures 7D,E), indicating no fitness advantage of nitrate reduction *in vitro*. When we performed a competitive 1:1 inoculation *in vivo,* however, recovery of the GZP-Km mutant from bladder and kidneys was significantly reduced compared to the WT (Figure 7F). The concentration of GZP-Km mutant in urine was also lower than the WT. The CFU counts for the WT were 1.5–2 orders of magnitude higher than those of the mutant as determined by the geometric means. This result demonstrated a significant competitive disadvantage for the nitrate reduction null mutant *in vivo*. This implicates nitrate reduction as a bacterial fitness factor for colonization of the urinary tract by UPEC.

## Discussion

Access to various terminal electron acceptors profoundly affects bacterial adaptation processes, such as biofilm formation (Bjergbaek et al., 2006; Martín Rodríguez, 2016; Eberly et al., 2017). Here we show that exogenous nitrate modulates the formation of biofilm in the UPEC strain CFT073. Nitrate supplementation downregulates ECM biosynthesis in the WT strain, whereas mutant strains lacking the membrane-bound nitrate reductases NarGHJI and NarZYWV showed increased biofilm production. Our results demonstrate a physiological association between nitrate reduction and biofilm ECM biosynthesis, which appears to be a relatively conserved feature of clinical UPEC. We were also able to demonstrate that the ability to reduce nitrate is a pathogenic fitness factor for CFT073 during UPEC infection *in vivo*.

Our mutational analysis showed that nitrate-induced ECM biosynthesis is CsgD-mediated, with curli fimbriae being a major component. Moreover, we revealed a tight interplay between sensing and reduction of extracellular nitrate and the regulation of biofilm ECM production. This finding is supported by a recent study, showing multiple pathways controlling the regulation of curli biosynthesis in *E. coli*, including respiration and environmental sensing (Smith et al., 2017). Furthermore, we have shown that the response regulator NarL contributes to the transcriptional regulation of *csgD* and hierarchically downstream genes. The complexity of the regulatory mechanisms, which could be direct or derived from pleiotropic effects, requires further molecular investigation to be fully elucidated.

Our finding of a link between nitrate sensing, respiration, and metabolism in biofilm formation dynamics is, to the best of our knowledge, unprecedented in *E. coli*. In *Pseudomonas aeruginosa,* nitrate metabolism was shown to affect flagellar motility, biofilm formation, and virulence factor production, with a central role of the NarL homologue (Van Alst et al., 2007). Nitrate reduction was also implicated as important for the structure of the *P. aeruginosa* colony biofilms by contributing to redox balancing (Dietrich et al., 2013). Our study in *E. coli* CFT073 thus provides an integrated physiological perspective that inter-connects the perception of environmental cues with cellular respiration and the CsgD regulatory network.

Previous research in tissue microbiology has shown that during infection, the complex microenvironment changes rapidly and dramatically (Boekel et al., 2011; Richter-Dahlfors et al., 2012). We have shown that the oxygen concentration in the kidney drops quickly after infection, due in part to a protective local vascular coagulation response (Melican et al., 2008; Schulz et al., 2018). From the microbial physiology standpoint, the altered local tissue environment during infection requires adaptation that profoundly affects the bacterial lifestyle. In this work, we demonstrated that nitrate reduction provides a competitive fitness advantage during UTI *in vivo,* meaning that bacteria without the ability to reduce nitrate are less able to effectively colonize the urinary tract when in competition. The fitness disadvantage of the nitrate respiration null mutant may not be related to differences in biofilm formation *in vivo* but derived from the inability to make use of this electron acceptor during UTI. Nitrate is naturally occurring in urine, with levels varying dependent on dietary uptake, and it can also be generated *in situ* as a consequence of local inflammation (Poljakovic et al., 2001). UPEC isolated from the urine of UTI patients has shown the ability to utilize aerobic or anaerobic respiration dependent on oxygen and nitrate levels in the urine (Hagan et al., 2010). The nitrate transporter *narK* has been identified as a fitness factor during experimental murine UTI (Buckles et al., 2015). The expression of several nitrate metabolism genes, including nitrate reductases, transporters, and response regulators, has been recently shown to be affected by the transcriptional regulator TosR that controls the expression of the non-fimbrial adhesion TosA in *E. coli* CFT073 (Luterbach et al., 2018). Other *E. coli* subspecies such as enteric *E. coli* in the intestinal tract have been shown to use host-derived nitrate to proliferate in the inflamed intestinal lumen (Spees et al., 2013; Winter et al., 2013). Consistently, commensal *E. coli* unable to reduce nitrate showed defects in the colonization of the mouse intestine (Jones et al., 2011). Our findings support a hypothesis in which the ability to reduce nitrate provides a fitness advantage for UPEC, aiding bacterial proliferation and dissemination within the urinary tract.

Collectively, our work provides an integrated perspective on the impact of nitrate reduction on UPEC physiology *in vitro* and *in vivo.* The suggested role of NarL as a key regulator linking environmental nitrate sensing and respiration with biofilm formation illustrates the complex regulatory networks that enable bacterial adaptation to dynamic environmental conditions. These insights establish nitrate respiration as key to triggering a lifestyle switch in *E. coli* and as a potential contributor to the *in vivo* pathogenesis of UTI.

## Data Availability Statement

The datasets generated for this study are available on request to the corresponding author.

## Ethics Statement

The animal study was reviewed and approved by Stockholms Norra Djurförsöksetiska Nämnd.

## Author Contributions

AM-R, MR, KM, and AR-D contributed to study design. AM-R performed the experiments. KM assisted the *in vivo* experiments. AM-R, MR, KM, and AR-D analyzed the data. AM-R, MR, KM, and AR-D wrote the manuscript.

### Conflict of Interest

The authors declare that the research was conducted in the absence of any commercial or financial relationships that could be construed as a potential conflict of interest.
