# Supplementary material for: Nitrate Metabolism Modulates Biosynthesis of Biofilm Components in Uropathogenic Escherichia coli and Acts as a Fitness Factor During Experimental Urinary Tract Infection
Source: Front Microbiol. 2020 Jan 31;11:26. doi: 10.3389/fmicb.2020.00026 (PMC7005491; doi:10.3389/fmicb.2020.00026)
Supplement: Supplementary file 1 [file Data_Sheet_1.docx]

**Nitrate Metabolism Modulates Biosynthesis of Biofilm Components in Uropathogenic *Escherichia coli* and Acts as a Fitness Factor During Experimental Urinary Tract Infection**

Alberto J. Martín-Rodríguez, Mikael Rhen, Keira Melican and

Agneta Richter-Dahlfors

**SUPPLEMENTARY MATERIAL**

**Figure S1** Colony biofilm phenotypes of 40 UPEC isolates in the absence (-) or presence (+) of 20 mM nitrate on LBNS CR agar after 48 h of incubation at 28 °C. A decrease in the red, dry and rough (*rdar*) morphotype upon nitrate addition is represented by a downwards green arrow, whereas an increase in the *rdar* morphotype is represented by an upwards red arrow. A black equal symbol indicates no relevant phenotypic change upon nitrate supplementation.

**
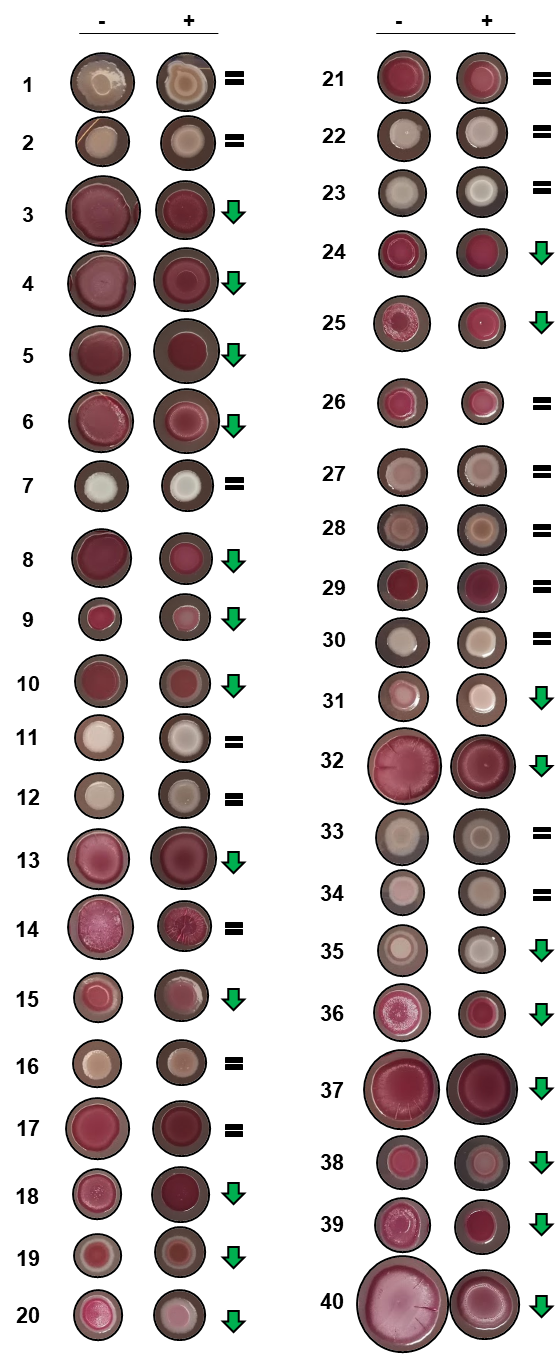
**

**Figure S2.** Colony morphotypes of *E. coli* CFT073 WT, [WT] Δ*bcsA*::Km, [WT] Δ*csgBAC*::Km, GZP; [GZP] Δ*bcsA*::Km and [GZP] Δ*csgBAC*::Km on calcofluor-supplemented LBNS agar plates with (A) or without 20 mM nitrate (B) after 48 h of incubation at 28 ºC, revealed upon exposure to UV-light.


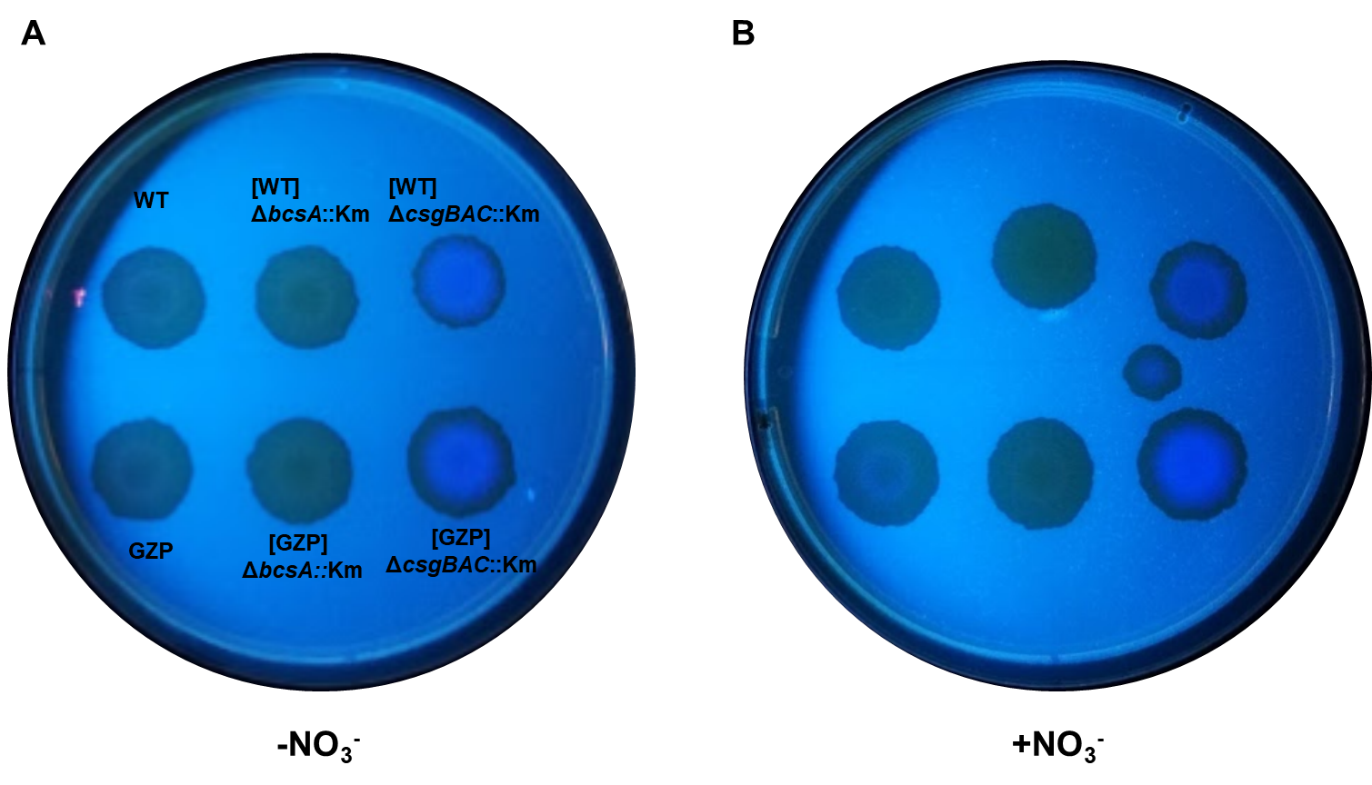


**Figure S3** Colony morphotypes (LBNS, 28 ºC, 48 h) after complementation of *narL* gene deletions in the WT and nitrate reductase null strain backgrounds in the absence (-) or presence (+) of 20 mM nitrate.


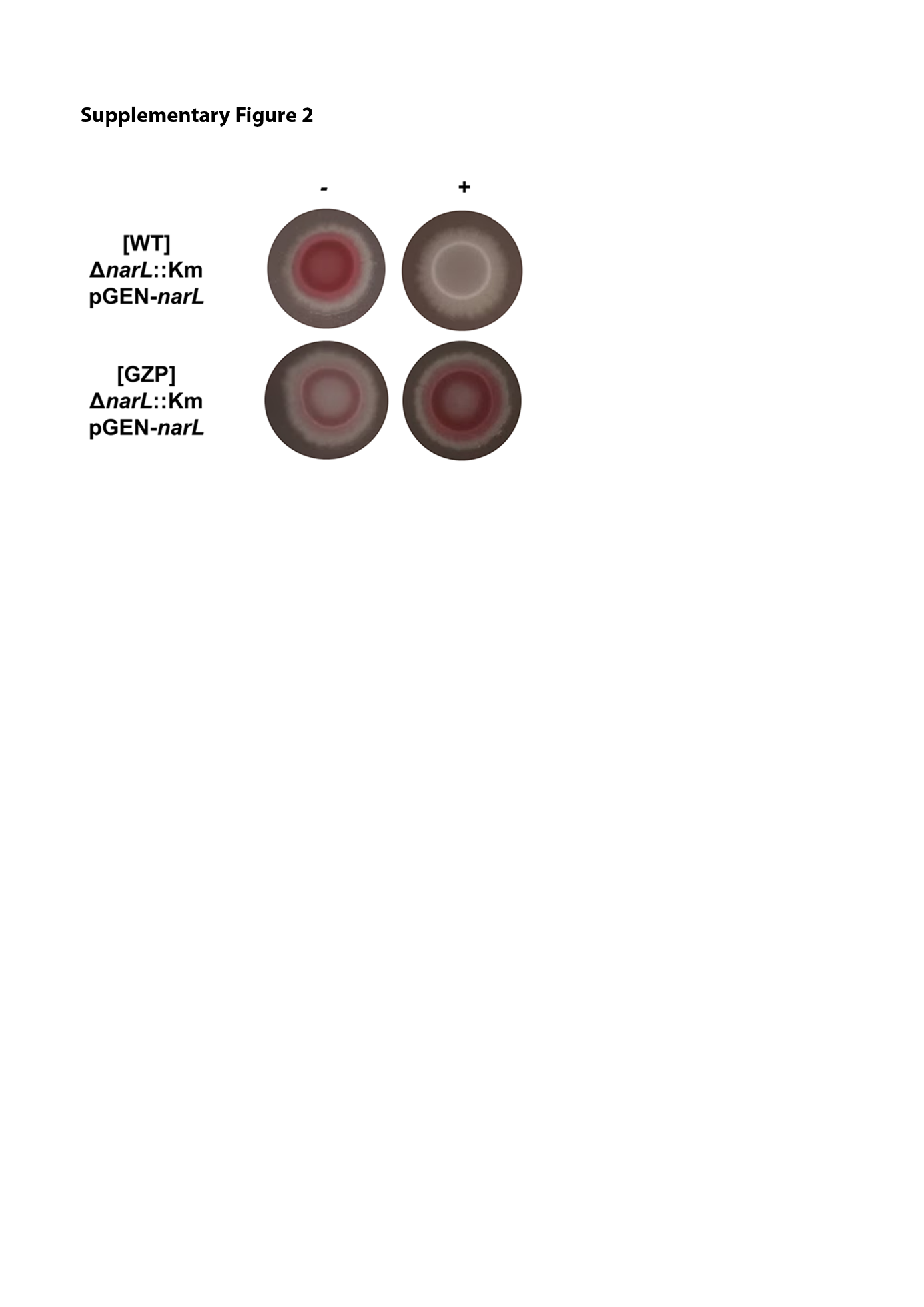


**Figure S4** Expression of *bcsA*, encoding the cellulose synthase, in colony biofilms of WT and nitrate reduction null mutant GZP in the absence or presence of 20 mM nitrate. Statistical significance was determined by one-way ANOVA followed by Dunnett’s post-hoc test (ns = P > 0.05).


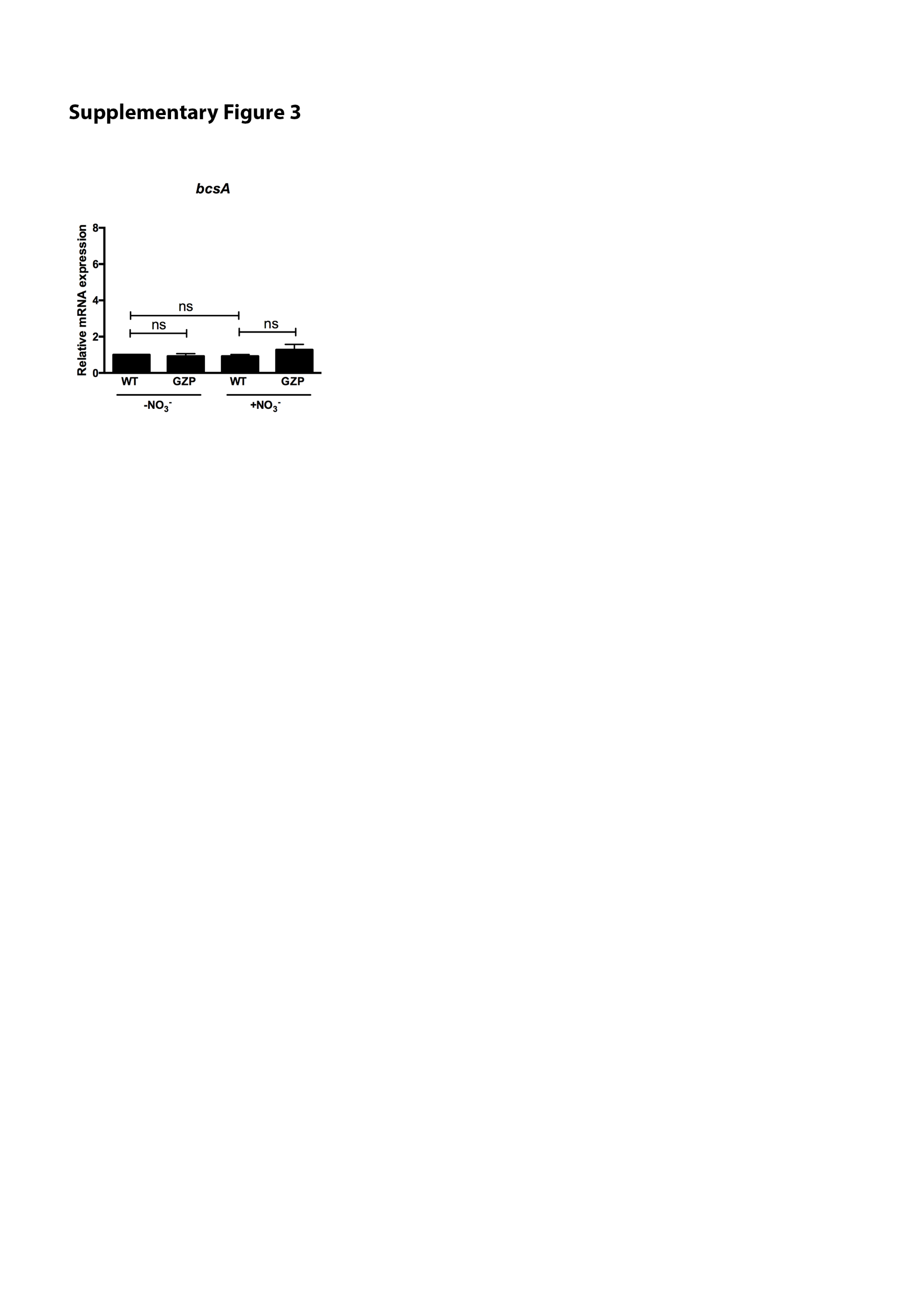


**Figure S5** Phenotypes of *csgD*:3XFLAG-tagged strains in the WT and GZP genetic backgrounds. Each strain is tested in quadruplicate in the absence (0) and presence of growing concentrations (0.2 mM, 2 mM, and 20 mM) of nitrate after incubation at 28°C for 24 h and 48 h.


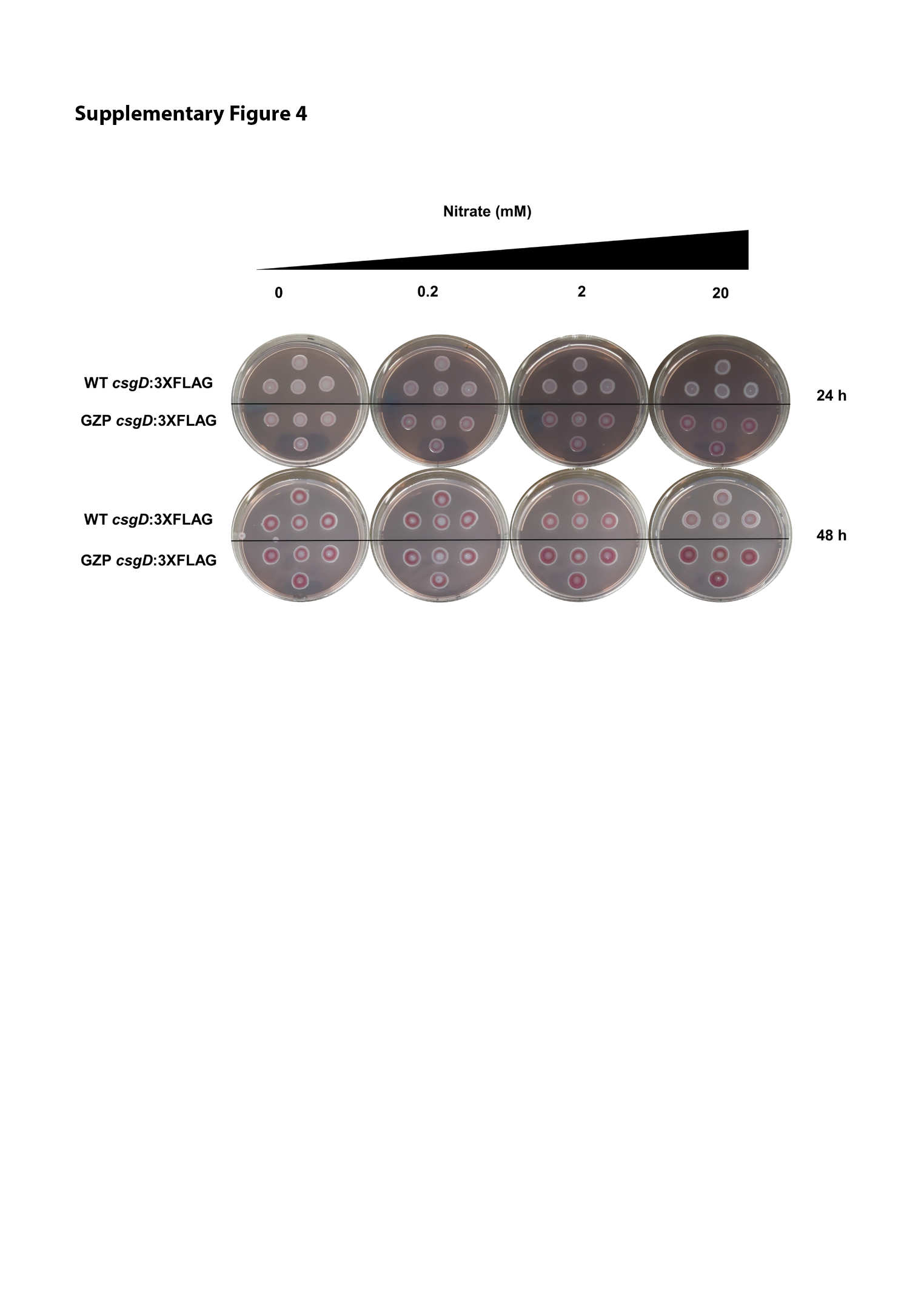


**Figure S6** Growth curve of (A) *E. coli* CFT073 WT, (B) GZP mutant and (C) overlay in LB medium over 24 h (N = 6).


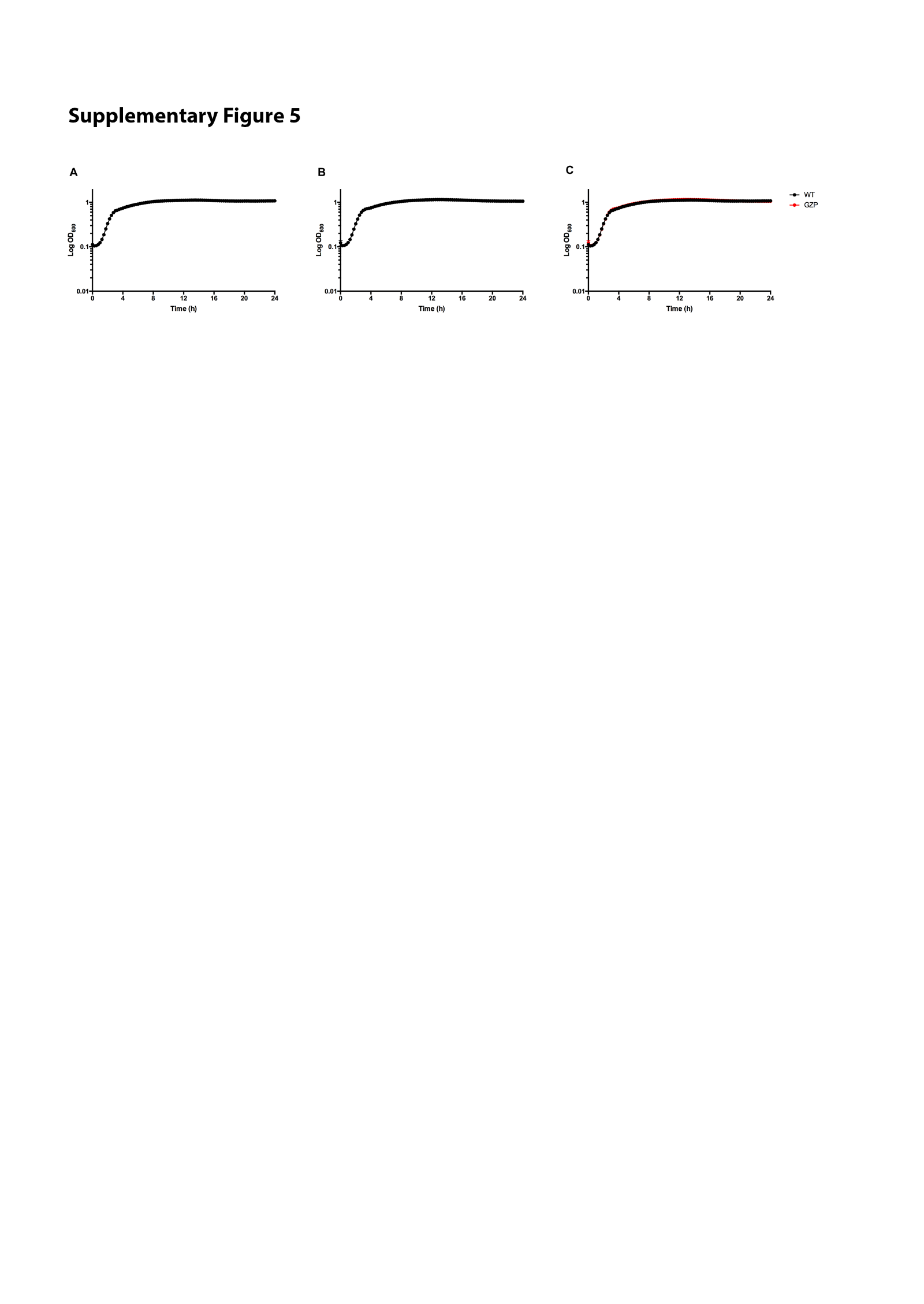


**Table S1** Strains used in this study.

| **Strain** | **Description** | **Source or reference** |
| --- | --- | --- |
| **Cloning strain** | | |
| *Escherichia coli* TOP10 | F^−^, *mcrA*, Δ(*mrr*-*hsdRMS*-*mcrBC*), φ80, *lacZ*ΔM15, Δ*lacX*74, *deoR*, *nupG*, *recA1*, *araD139*, Δ(*ara*-*leu*)7697, *galU*, *galK*, *rpsL* (Str^R^), *endA1* | Invitrogen |
| ***Escherichia coli* CFT073 strains** | | |
| *Escherichia coli* CFT073 | Pyelonephritis isolate, wild-type strain | (1) |
| G | [WT] Δ*narGHJI* | This study |
| Z | [WT] Δ*narZYWV* | This study |
| P | [WT] Δ*napFDAGHBC* | This study |
| GZ | [WT] Δ*narGHJI* Δ*narZYWV* | This study |
| GP | [WT] Δ*narGHJI* Δ*napFDAGHBC* | This study |
| ZP | [WT] Δ*narZYWV* Δ*napFDAGHBC* | This study |
| GZP | [WT] Δ*narGHJI* Δ*narZYWV* Δ*napFDAGHBC* = [GZP] | This study |
| GZP-Km | [WT] Δ*narGHJI*::Km Δ*narZYWV* Δ*napFDAGHBC* | This study |
| WT-*bcsA^-^* | [WT] Δ*bcsA*::Km | This study |
| GZP-*bcsA^-^* | [GZP] Δ*bcsA*::Km | This study |
| WT-*yaiC^-^* | [WT] Δ*yaiC*::Km | This study |
| GZP-*yaiC^-^* | [GZP] Δ*yaiC*::Km | This study |
| WT-*csgBAC^-^* | [WT] Δ*csgBAC*::Km | This study |
| GZP-*csgBAC^-^* | [GZP] Δ*csgBAC*::Km | This study |
| WT-*csgD*^-^ | [WT] Δ*csgD* | This study |
| GZP-*csgD*^-^ | [GZP] Δ*csgD* | This study |
| WT-*mlrA^-^* | [WT] Δ*mlrA*::Km | This study |
| GZP-*mlrA^-^* | [GZP] Δ*mlrA*::Km | This study |
| WT-*rpoS^-^* | [WT] Δ*rpoS*::Km | This study |
| GZP-*rpoS^-^* | [GZP] Δ*rpoS*::Km | This study |
| WT-*ydaM^-^* | [WT] Δ*ydaM*::Km | This study |
| GZP-*ydaM^-^* | [GZP] Δ*ydaM*::Km | This study |
| WT-*narU^-^* | [WT] Δ*narU*::Km | This study |
| GZP-*narU^-^* | [GZP] Δ*narU*::Km | This study |
| WT-*narK^-^* | [WT] Δ*narK*::Km | This study |
| GZP-*narK^-^* | [GZP] Δ*narK*::Km | This study |
| WT-*narX^-^* | [WT] Δ*narX*::Km | This study |
| GZP-*narX^-^* | [GZP] Δ*narX*::Km | This study |
| WT-*narQ^-^* | [WT] Δ*narQ*::Km | This study |
| GZP-*narQ^-^* | [GZP] Δ*narQ*::Km | This study |
| WT-*narP^-^* | [WT] Δ*narP*::Km | This study |
| GZP-*narP^-^* | [GZP] Δ*narP*::Km | This study |
| WT-*narL^-^* | [WT] Δ*narL*::Km | This study |
| GZP-*narL^-^* | [GZP] Δ*narL*::Km | This study |
| WT/CsgD-FLAG | [WT] *csgD*::3XFLAG | This study |
| GZP/CsgD-FLAG | [GZP] *csgD*::3XFLAG | This study |
| WT-*narL*^-^/CsgD-FLAG | [WT] Δ*narL*::Km *csgD*-3XFLAG | This study |
| GZP-*narL*^-^/CsgD-FLAG | [GZP] Δ*narL*::Km *csgD*-3XFLAG | This study |
| ***Escherichia coli* CFT073 complemented strains and vector controls** | | |
| *Escherichia coli* CFT073-VC | Wild-type strain with pGEN-MCS | This study |
| G-VC | [WT] Δ*narGHJI* with pGEN-MCS | This study |
| Z-VC | [WT] Δ*narZYWV* with pGEN-MCS | This study |
| P-VC | [WT] Δ*napFDAGHBC* with pGEN-MCS | This study |
| GZ-VC | [WT] Δ*narGHJI* Δ*narZYWV* with pGEN-MCS | This study |
| GP-VC | [WT] Δ*narGHJI* Δ*napFDAGHBC* with pGEN-MCS | This study |
| PZ-VC | [WT] Δ*narZYWV* Δ*napFDAGHBC* with pGEN-MCS | This study |
| GZP-VC | [GZP] with pGEN-MCS | This study |
| G-pGEN-G | [WT] Δ*narGHJI* with pGEN-G | This study |
| Z-pGEN-Z | [WT] Δ*narZYWV* with pGEN-Z | This study |
| P-pGEN-P | [WT] Δ*napFDAGHBC* with pGEN-P | This study |
| GP-pGEN-GP | [WT] Δ*narGHJI* Δ*napFDAGHBC* with pGEN-GP | This study |
| PZ-pGEN-PZ | [WT] Δ*narGHJI* Δ*narZYWV* with pGEN-ZP | This study |
| GZ-pGEN-GZ | [WT] Δ*narGHJI* Δ*narZYWV* with pGEN-GZ | This study |
| GZP-pGEN-GZP | [GZP] with pGEN-GZP | This study |
| WT-*narL*^-^-pGEN-*narL* | [WT] Δ*narL*::Km with pGEN-*narL* | This study |
| GZP-*narL*^-^-pGEN-*narL* | [GZP] Δ*narL*::Km with pGEN-*narL* | This study |
| **Other *Escherichia coli* CFT073 strains** | | |
| WT (pBAD/Myc-His) | WT with pBAD/Myc-His | This study |
| GZP (pBAD/Myc-His) | GZP with pBAD/Myc-His | This study |
| WT (pBAD-*slr1143*) | WT with pBAD-*slr1143* | This study |
| GZP (pBAD-*slr1143*) | GZP with pBAD-*slr1143* | This study |
| WT (pBAD-*yhjH*) | [WT] with pBAD-*yhjH* | This study |
| GZP (pBAD-*yhjH*) | [GZP] with pBAD-*yhjH* | This study |
| WT (pRP0122-P*be*-*amcyan*_*Bc3*-*5*_*turborfp*) | [WT] with pRP0122-P*be*-*amcyan*_*Bc3*-*5*_*turborfp* | This study |
| GZP (pRP0122-P*be*-*amcyan*_*Bc3*-*5*_*turborfp*) | [GZP] with pRP0122-P*be*-*amcyan*_*Bc3*-*5*_*turborfp* | This study |

(1) **Kao JS**, **Stucker DM**, **Warren JW**, **Mobley HL**. 1997. Pathogenicity island sequences of pyelonephritogenic *Escherichia coli* CFT073 are associated with virulent uropathogenic strains. Infect Immun **65**:2812–20.

**Table S2** Plasmids and primers used in this study. Recombination sites are shown in bold and cursive. Restriction sites appear in bold and underlined.

| **Plasmid or primer** | **Description or sequence (5’🡪3’)** | **Source or reference** |
| --- | --- | --- |
| **Plasmids** | | |
| pKD4 | *bla* FRT *kan* FRT oriR6K (Km^R^, Amp^R^) | (1) |
| pSIM6 | pSC101 ori^TS^ P_L_-*gam-bet-exo cI*857 (Amp^R^) | (2) |
| pCP20 | pSC101 derivative; *repA101*^TS^ *FLP*+ λ*cI*857 λ*p*_R_; (Amp^R^, Cm^R^) | (1) |
| pSUB11 | R6KoriV bla 3×flag-FRT-kan-FRT (Amp^R^) | (3) |
| pGEN-MCS | pGEN222 derivative, *gfpuv* repaced by a MCS after digestion with EcoRI and SalI, *bla* (Amp^R^) | Addgene |
| pBAD/Myc-His | *bla*, pBR322 *ori*, P*_ARA_*::*myc-His_6_*^+^ (Amp^R^) | Addgene |
| pRP0122-P*be*-*amcyan*_*Bc3*-*5*_*turborfp* | pSC101 derivative; P*be*, *speR*, *amcyan*-*bc345*-*turporfp*. Cyclic di-GMP reporter. | (4) |
| pGEN-G | pGEN::*narGHJI*; *narGHJI* operon and its 500 bp upstream region containing the native promoter cloned in pGEN-MCS | This study |
| pGEN-Z | pGEN::*narZYWV*; *narZYWV* operon and its 500 bp upstream region containing the native promoter cloned in pGEN-MCS | This study |
| pGEN-P | pGEN::*napFDAGHBC*; *napFDAGHBC* operon and its 500 bp upstream region containing the native promoter cloned in pGEN-MCS | This study |
| pGEN-GZ | pGEN::*narGHJI*::*narZYWV*; *narGHJI* and *narZYWV* operons and their 500 bp upstream regions containing their respective native promoters cloned in tandem in pGEN-MCS | This study |
| pGEN-GP | pGEN::*narGHJI*::*napFDAGHBC*; *narGHJI* and *napFDAGHBC* operons and their 500 bp upstream regions containing their respective native promoters cloned in tandem in pGEN-MCS | This study |
| pGEN-PZ | pGEN::*narZYWV*::*napFDAGHBC*; *narZYWV* and *napFDAGHBC* operons and their 500 bp upstream regions containing their respective native promoters cloned in tandem in pGEN-MCS | This study |
| pGEN-GZP | pGEN::*narGHJI*::*narZYWV*::*napFDAGHBC*; *narGHJI*, *narZYWV* and *napFDAGHBC* operons and their 500 bp upstream regions containing their respective native promoters cloned in tandem in pGEN-MCS | This study |
| pGEN-*narL* | pGEN::*narL*; *narL* gene and its 535 upstream region containing the native promoter cloned in pGEN-MCS | This study |
| pBAD-*slr1143* | pBAD-Myc-His::*slr1143* | This study |
| pBAD-*yhjH* | pBAD-Myc-His::*yhjH* | This study |
| **Primers for λ-red recombineering** | | |
| Lambda_*narGHJI*-F | AGCAATGTCGATTTATCAGAGGGCCGACAGGCTCCCACAGGAGAAAACCG***GTGTAGGCTGGAGCTGCTTC*** | This study |
| Lambda_*narGHJI*-R | ACAATCCGAAAAATCGGCAGTGTGTAACCACCGATGTTTGGTTTAGATAA***CATATGAATATCCTCCTTAG*** | This study |
| Lambda_*napFDAGHBC*-F | TTATTTGGTCGCTCTCAATTTTCAGAGCGCGTTAATGATGGAAGGTCAAT***GTGTAGGCTGGAGCTGCTTC*** | This study |
| Lambda_*napFDAGHBC*-R | CGCTCGCAAAGTAACTCTCTGGCTTCAAGCATACCCACGCAATAACCCTG***CATATGAATATCCTCCTTAG*** | This study |
| Lambda_*narZYWV*-F | AGTGTTTTTAATCTTCTACATCGTTTGTGTGCTACTGACCTGGCTGATTT***GTGTAGGCTGGAGCTGCTTC*** | This study |
| Lambda_*narZYWV*-R | ATAGCCTGCAAGTGGCCGGAGAGCGAAGGGCTATCCGGCCCGCGTGAGAA***CATATGAATATCCTCCTTAG*** | This study |
| Lambda-*bcsA*-F | TGGTGCCTGTTAAACTATTCCGGGCTGAAAACGCCAGTCGGGAGTGCATC***GTGTAGGCTGGAGCTGCTTC*** | This study |
| Lambda-*bcsA*-R | CCAGAGCCACTGCACAAATCCAGAATATTTTTCTTTTCATCGCGTTATCA***CATATGAATATCCTCCTTAG*** | This study |
| Lambda_*yaiC*-F | ATACTTCTGCCTTTAGCTCCGTCTCTATAATTTGGGAAAATTGTTTCTGA***GTGTAGGCTGGAGCTGCTTC*** | This study |
| Lambda_*yaiC*-R | TATGGAAAAATCAGAAAAACTCAGCAAATCCTGATGACTTTCGCCGGACG***CATATGAATATCCTCCTTAG*** | This study |
| Lambda-*csgBAC*-F | CAAAATACAGGTTGCGTTAACAACCAAGTTGAAATGATTTAATTTCTTAA***GTGTAGGCTGGAGCTGCTTC*** | This study |
| Lambda-*csgBAC*-R | GCAGCAGACCATTCTCTCCAGGTTCATCTTATGCTCGATATTTCAACAAA***CATATGAATATCCTCCTTAG*** | This study |
| Lambda-*csgD*-F | TTAGAGGCAGCTGTCAGGTGTGCGATCAATAAAAAAAGCGGGGTTTCATC***GTGTAGGCTGGAGCTGCTTC*** | This study |
| Lambda-*csgD*-R | CGAACAGAAATTCTGCCGCCACAATCCAGCGTAAATAACGTTTCATGGCT***CATATGAATATCCTCCTTAG*** | This study |
| Lambda-*mlrA*-F | CAAAACTGCGTCTAAAGTTAAACCGGGACCTCGCGAGCAAGGGTGAGACG***GTGTAGGCTGGAGCTGCTTC*** | This study |
| Lambda-*mlrA*-R | TTATGTTAACGAAAGGATTGTACAGTAAAGCGCATTTGTTAACGAATCAT***CATATGAATATCCTCCTTAG*** | This study |
| Lambda-*rpoS*-F | aggcttttgcTTGAATGTTCCGTCAAGGGATCACGGGTAGGAGCCACCTT***GTGTAGGCTGGAGCTGCTTC*** | This study |
| Lambda-*rpoS*-R | agaaaaaaagCCAGCCTCGCTTGAGACTGGCCTTTCTGACAGATGCTTAC***CATATGAATATCCTCCTTAG*** | This study |
| Lambda-*ydaM*-F | CCGTGTGAAGTGTTAAATAGCGTCTATCATTATCAGAATTATCTGATCAT***GTGTAGGCTGGAGCTGCTTC*** | This study |
| Lambda-*ydaM*-R | TGGGAGCGCCACGCTAAGCGCAGTCGTTGATCTCGAGACGCATCCGCGGC***CATATGAATATCCTCCTTAG*** | This study |
| Lambda_*narU*-F | TCGGATGAGACACGGTTGTAGCCTGATAAGACGTGTCAGGCATCGATCTC***GTGTAGGCTGGAGCTGCTTC*** | This study |
| Lambda_*narU*-R | CTCCTGTTTTGTCTTTTTTTCATTACCAATGTGTGCATGTGAGGAACAAT***CATATGAATATCCTCCTTAG*** | This study |
| Lambda_*narK*-F | GCCTTTAGCTACAGACACTAAGGTGGCAGACATCGAAACGAGTATCAGAG***GTGTAGGCTGGAGCTGCTTC*** | This study |
| Lambda_*narK*-R | TAAAAAAAGCGCGACATCATGCCGCGCAAAGGATAATCAAATATTACGAT***CATATGAATATCCTCCTTAG*** | This study |
| Lambda_*narX*-F | CACATTCATTAAGGTTATTGCTCATTTAAAGCCTGAAGGAAGAGGTTTAC***GTGTAGGCTGGAGCTGCTTC*** | This study |
| Lambda_*narX*-R | TTCGCAACATCGGGTGATCGTCGATCAGCAGGATAGTAGCCGGTTCCTGA***CATATGAATATCCTCCTTAG*** | This study |
| Lambda_*narQ*-F | CATCACTGACTAAATTTCGTTTCAGCGAACTGGAACATTAATGATTTTTT***GTGTAGGCTGGAGCTGCTTC*** | This study |
| Lambda_*narQ*-R | CCTTTTATACTGAACCTTAAGTGCCACTATTCTTTGGTCAATAAGAGGCA***CATATGAATATCCTCCTTAG*** | This study |
| Lambda_*narP*-F | GGGTAACATTCACGCGCCTGGTAGCGTTACCAACGCTACGCTCAAACATA***GTGTAGGCTGGAGCTGCTTC*** | This study |
| Lambda_*narP*-R | GCAGGAGAGTAAATAAAAAGTGGCCCGATGGTGATGCCATCGGGCTATTT***CATATGAATATCCTCCTTAG*** | This study |
| Lambda_*narL*-F | CCGTGCGCAAAGTTTACGAGGCGATTGCCGCGTCCGCCGTCGTGAATCAG***GTGTAGGCTGGAGCTGCTTC*** | This study |
| Lambda_*narL*-R | TCGAACCGTTCGACGCATTGTCAAACGACGAACTGCGCTGGGAACCGTAA***CATATGAATATCCTCCTTAG*** | This study |
| **Primers for *csgD* epitope tagging** | | |
| *csgD*-Epit-F | GACACAAGCGGTTTCCTGGGCAAACGATAACCTCAGGCGA***GACTACAAAGACCATGACGG*** | This study |
| *csgD*-Epit-R | GCCACAATCCAGCGTAAATAACGTTTCATGGCTTTATCGC***CATATGAATATCCTCCTTAG*** | This study |
| **Primers for mapping gene disruptions** | | |
| Check_*narGHJI*-F | CGTTATCAATTCCCACGCTGT | This study |
| Check_*narGHJI*-R | CTTCACTGCTCCATAGGTGGA | This study |
| Check_*narZYWV*-F | GCGTTTGGCATGTCGCTC | This study |
| Check_*narZYWV*-R | GTGGTAAACCTGCGGTTTCA | This study |
| Check_*napFDAGHBC*-F | ATCTTAGCGGCTATAAAAATGGC | This study |
| Check_*napFDAGHBC*-R | TGCGTTCAGCGTAAATGACAAG | This study |
| Check_*bcsA*-F | AATTTCGCAGTGATGCGCT | This study |
| Check_*bcsA*-R | GTCGCCTGCGTCATGAAAGA | This study |
| Check_*yaiC*-F | CACAACAATCTCTGAAAGGTGA | This study |
| Check_*yaiC*-R | GGGTTCCGCAAAAATGGT | This study |
| Check_*csgBAC*-F | TCCGCAGACATACTTTCCAT | This study |
| Check_*csgBAC*-R | CCCATACTTTCAGGTTGGCT | This study |
| Check_*csgD*-F | TTACTACACACAGCAGTGCAAC | This study |
| Check_*csgD*-R | AGTTAACAATCCCGGGACTTCT | This study |
| Check_*mlrA*-F | TTAAAACGCGTAACATACATTGC | This study |
| Check_*mlrA*-R | CAGACCGCCTGTTTGAAAG | This study |
| Check_*rpoS*-F | TAATGATGATTACCTGAGTGCCTA | This study |
| Check_*rpoS-*R | CCTACGGGCGATCTGGATA | This study |
| Check_*ydaM*-F | TTCCACAGTGCCTCCATCAT | This study |
| Check_*ydaM*-R | AATATTGTCCGCAGCTATGTG | This study |
| Check_*narU*-F | GTTGCTATGCATCACCTGTCC | This study |
| Check_*narU*-R | CTGTCTGTTTAATCCACATTCTGCC | This study |
| Check_*narK*-F | ATTGGCTGAAGTATACCCATACCC | This study |
| Check_*narK*-R | TGGGTATTAAGGAGTATTCCCCA | This study |
| Check_*narX*-F | CACGGTACTGTAAAATCCCTACC | This study |
| Check_*narX*-R | AGTTTATCCAGCGTTTCCAGACC | This study |
| Check_*narQ*-F | GGCGATTTCACAAGCATGAC | This study |
| Check_*narQ*-R | CCATTAAAAGAGGACCTCGTG | This study |
| Check_*narP*-F | TTCCTGATGAGAAGCTGATGC | This study |
| Check_*narP*-R | ATAGCAATATCCGTGTGTTAAGC | This study |
| Check_*narL*-F | ACTGACCGTCCAGGATAACG | This study |
| Check_*narL*-R | CGCACAAGCCAACAGTGAG | This study |
| **qRT-PCR primers** | | |
| q-*fliC*-F | AATTCAGGTTGGTGCGAACG | This study |
| q-*fliC*-R | AATGGTCGCCGCTTTATTGG | This study |
| q-*csgD*-F | ACCGCGACATTGAAAACTGG | This study |
| q-*csgD*-R | TGCAACCCATTGACAACACG | This study |
| q-*csgA*-F | TGATCTGACCATTACCCAGCAC | This study |
| q-*csgA*-R | ATCAAGAGTAGCGCTGTTGC | This study |
| q-*rpoS*-F | AAAAGCGTTGCTGGACATCC | This study |
| q-*rpoS*-R | ACAGCCATTTGACGATGCTC | This study |
| q-*narL*-F | AGTTCACGTCAAGCACATGC | This study |
| q-*narL*-R | AAAATGCGCTCCTGATGCAC | This study |
| q-*rpoD*-F | ATGGTTGAAGCGAACTTGCG | This study |
| q-*rpoD*-R | TCAACCGCTTTCATCAGACC | This study |
| **Cloning primers** | | |
| Compl-*narGHJI*-F-BmtI | AT**GCTAGC**CTGGCATTAACGGGTTCGCC | This study |
| Compl-*narGHJI*-R-SacI | ATC**GAGCTC**TTAGTGACGGGCGCGCAC | This study |
| Compl-*narZYWV*-F-SacI | CTA**GAGCTC**TATTCTGCGCCTGGCGTTCTT | This study |
| Compl-*narZYWV*-R-KpnI | ATA**GGTACC**TTAACGCCGCGAACGCACA | This study |
| Compl-*napFDAGHBC*-F-KpnI | ATA**GGTACC**TGCGATTTTTTCCAGTGGCT | This study |
| Compl-*napFDAGHBC*-R-SbfI | AT**CCTGCAGG**TTAAAAACCTGGCTCGACTT | This study |
| Compl-*narL*-F-KpnI | ATA**GGTACC**GAACTGCATGATTCTATTGCCCA | This study |
| Compl-*narL*-R-SalI | ATA**GTCGAC**TCAGAAAATGCGCTCCTGATG | This study |
| Slr_HindIII-F | ATA**AAGCTT**ATGGAAGCTAAATTACCGCA | This study |
| Slr_XbaI-F | A**TCTAGA**TTATTCTGCCAGTTGAAAATTG | This study |
| CFyhjH_HindIII-F | CTA**AAGCTT**TTGATGATAAGGCAGGTTAT | This study |
| CFyhjH_XbaI-R | ATA**TCTAGA**TTATAGCGCCAGAACCGC | This study |
